# Psychometric properties of the *Stress Mindset Measure* (SMM) in the Polish population

**DOI:** 10.1371/journal.pone.0264853

**Published:** 2022-03-22

**Authors:** Dorota Mierzejewska-Floreani, Mateusz Banaszkiewicz, Ewa Gruszczyńska

**Affiliations:** SWPS University of Social Sciences and Humanities, Warsaw, Poland; Aalborg University, DENMARK

## Abstract

**Purpose:**

The goal of the research was to evaluate the psychometric properties of the Polish adaptation of the *Stress Mindset Measure* (SMM; general version, SMM-G, and specific version, SMM-S).

**Methods:**

Study 1 was an online survey conducted among 1651 adults (81% women, aged 18–84 years). To assess the theoretical validity of the SMM, the following constructs were also measured: Big Five personality dimensions, positive orientation, self-control, perceived stress at work, depressiveness, assessment of one’s own health, and ego-resiliency. Study 2 was a test-retest reliability measurement and took place 10 months later among 344 participants.

**Results:**

A factor validity was examined using exploratory (EFA) and confirmatory (CFA) factor analysis. EFA revealed a two-factor structure for the SMM-G and a one-factor structure for the SMM-S. However, these models obtained unsatisfactory goodness-of-fit indices in the CFA. Among the alternative models, the four-factor hierarchical model was best fitted to the data for both the SMM-G (RMSEA = .038, CFI = .996, TLI = .985) and the SMM-S (RMSEA = .041, CFI = .996, TLI = .990). These results were supported in the test-retest sample (SMM-G: RMSEA = .066, CFI = .990, TLI = .968; SMM-S: RMSEA = .056, CFI = .994, TLI = .983). Thus, four lower-order factors were identified: General, Health and Vitality, Performance and Productivity, Learning and Growth. The reliability of the overall general and specific indices measured with Cronbach’s alpha was high and repeatable in both studies (Study 1: SMM-G α = .88; SMM-S α = .91; Study 2 (SMM-G, α = .87; SMM-S, α = .91). The stability for the SMM-G was satisfactory (r = .62; *p* < .001), and moderate for SMM-S (r = .46, *p* < .001). The theoretical validity analysis showed low (< |.40|) correlations in the expected directions with the majority of the selected tools.

**Conclusion:**

The Polish adaptation of the SMM has very good psychometric properties. However, the unidimensional character of the original scale is not confirmed, which was also the case in other existing adaptations. The analyses in a sample several times larger than in previous studies revealed a greater complexity of the construct, identifying one higher-order factor and four lower-order factors.

## Introduction

The belief that stress is harmful is so widely held that it can become harmful in itself [1]. In fact, there are a number of data indicating much greater variation in the outcomes of experiencing stress, both situationally and interpersonally [2]. Thus, the course and consequences of a stressful situation can be influenced not only by how a person perceives it at a given time, but also by how this person generally assesses the role of stress in their life. Attention is drawn to the possible importance of a generalized stress mindset by research on core beliefs and their role in shaping cognitive, emotional, and behavioral responses.

### Stress mindset

A mindset is understood as “a mental frame or lens that selectively organizes and encodes information, thereby orienting an individual toward a unique way of understanding an experience and guiding one toward corresponding actions and responses” [3, p. 91–394]. Thus, it is a construct in the cognitive area that is firmly integrated with the individual’s beliefs about the world. Accordingly, a stress mindset is a set of well-established beliefs about the nature of stress its consequences. Crum et al. [4] describe the stress mindset as the degree to which a person holds beliefs that stress has positive (*stress-is-enhancing mindset*) or negative (*stress-is-debilitating mindset*) consequences for stress-related outcomes connected with health and vitality as well as with performance and productivity, and learning and growth.

The stress mindset, which is considered to be composed of metacognitive beliefs about the nature of stress, is distinct from cognitive appraisal. A primary appraisal is the cognitive process that occurs when one is appraising whether an event is stressful and relevant to him or her, and a secondary appraisal—is an evaluation of the potential resources and requirements needed to go through a given stressful situation [5]. Meanwhile, the stress mindset is more generalized attitude towards stress. It contains beliefs about the nature of stress across contexts that do not have to be directly reflected in appraisal of each stressful situation [4]. For example, one may view a particular stressor (e.g., an impending deadline) as highly stressful but have a *stress-is-enhancing mindset* (i.e., believing that experiencing this kind of stress ultimately results in enhancing outcomes). Conversely, one may also appraise the impending deadline as highly stressful but may have a *stress-is-debilitating mindset* (i.e., expecting the stressor to debilitate health and vitality) [4]. A study by Horiuchi and colleagues [6] showed that the stress mindset is associated with psychological stress responses through coping strategies. They observed that emotional expression partially mediated the relationship between a *stress-is-debilitating mindset* and irritability-anger level. Emotional support seeking, cognitive reinterpretation, and problem-solving did not show such mediating effects. None of the coping strategies mediated the relationship between a *stress-is-enhancing mindset* and stress responses.

There is also a study showing that, while experiencing acute stress, participants with a positive stress mindset manifested more adaptive responses (feedback receptiveness and moderate cortisol reactivity) [4]. Similarly, in an online cross-sectional survey, Keech and colleagues [7] found indirect effects of stress mindsets on perceived stress and psychological wellbeing through somatic symptoms and proactive coping behaviors. For 134 police officers, perceived stress and psychological and physical wellbeing were directly predicted by stress mindsets. Relationships between the stress mindset and health-related outcomes were mediated by behaviors aimed at proactively meeting demands and perceived somatic symptoms [7].

Moreover, a positive stress mindset was induced by a targeted intervention and was associated with positive changes in self-reported job performance and health measures [4]. Another study investigated in an experimental design a role of the stress mindset for cognitive, emotional, and neuroendocrine responses in participants under stress. The participants’ attitudes toward stress were manipulated by inducing a positive or negative stress mindset before exposing them to social stress, eliciting threatening or challenging appraisals. A positive stress mindset was associated with more adaptive responses such as increased positive emotions, interpreted as a psychological marker of resilience, and greater dehydroepiandrosterone (DHEA) secretion, a proxy of physiological resilience [8].

In a diverse and large sample (*N* = 1343) of adolescents, Park and others [9] examined the relationship between negative life events, perceived distress, and self-control. The association between negative life events and distress was weaker for participants with a positive stress mindset. Stress mindset appeared to modify how participants react emotionally to objective stressor events [9].

### Measures of the stress mindset

Crum and colleagues proposed the eight-item *Stress Mindset Measure* (SMM) [4]. Its General version—SMM-G—measures the stress mindset as the extent to which the belief that stress-is-enhancing is endorsed. The specific version—SMM-S is identical to the SMM-G, except that it asks participants to respond to questions in relation to the primary source of stress in their life currently. Confirmatory factor analysis revealed an unidimensional structure of both SMM versions. Factor loadings of all items on a single factor were relatively high, all starting from .50, but not exceeding .80. The SMM-G and SMM-S had a good internal consistency: Cronbach’s alpha was .86; and 80 respectively [4]. Pearson correlations revealed that, while the SMM was significantly correlated in the expected direction with all measures related to stress (such as the Perceived Stress Scale [PPS-10] or the Revised Life Orientation Test [LOT-R], and also the severity of stress, stress amount etc.), these correlations were small to moderate (r < |.50|; p < .001), suggesting that the SMM is a distinct construct [4].

The SMM already has several national validations. The unidimensional structure of the scale was not confirmed by analyses performed in the Greek, and [10] Japanese [11] populations. In general, the Greek version of the SMM has satisfactory reliability and validity indexes. However, the *stress-is-enhancing* and *stress-is-debilitating mindsets* were found to be separate components rather than opposite poles of the same dimension of the stress mindset [10]. Similarly, analyses of the Japanese version revealed a two-factor structure with a strong negative correlation between the harmfulness of stress and usefulness of stress [11].

An innovative questionnaire for measuring stress mindset—*the Stress Control Mindset Measure SCMM*—was proposed by Keech and colleagues [12]. They suggested that the Crum’s SMM items [4] conceptualize the stress mindset as a belief in the stress-is-enhancing (i.e., a fixed mindset that stress has positive consequences) on one end of the spectrum, and a belief in the stress-is-debilitating (i.e., a fixed mindset that stress has negative consequences) on the other end, whereas mindset theory conceptualizes the spectrum as being from fixed to growth (i.e., stress can be enhancing). Moreover, they concluded that the individual is an active participant in harnessing the enhancing consequences of stress [13].

Meeting these conclusions, Keech and colleagues [12] designed a 15-item self-report measure that operationalizes the stress mindset as not only the extent to which an individual holds the belief that the consequences of stress can be enhancing, but also that the individual can use stress to experience these enhancing consequences. For example, Crum’s item: *Experiencing stress facilitates my learning and growth* [4] was changed to *You can use stress to facilitate your learning and growth* [12]. This approach considers the possibility of an individual actively influencing their own stress mindset and is also more in line with the idea of mindset itself [4]. The hierarchical four-factor structure of the SCMM based on such reformulation of the items had a satisfactory fit in Australian (χ2 = 122.25, *p* < .001, CFI = .974, TLI = .962, RMSEA = .057) and in British (χ2 = 158.35, *p* < .001, CFI = .949, TLI = .925, RMSEA = .075) samples. For both samples reliability, and convergent and discriminant validity, as well as concurrent validity was confirmed [12]. The specific version of the SCMM has not been tested.

## Materials and methods

The adaptation process began with a translation of the original English-language version of the SMM, followed by a pilot study. The ethical committee approval was then obtained and recruitment for the first study was conducted. Study 1 included a 40-minute online survey among 1651 participants from the Polish population. Study 2 took place 10 months later in a group of individuals who agreed to participate in the retest. Both data sets were then used in establishing the psychometric properties of the SMM. The factorial and theoretical validity were examined. Also, two types of reliability were assessed: internal consistency and stability.

### Translation and pilot study

The first stage entailed a translation of the questionnaire. After obtaining a written consent from the authors of the original, it was translated from an English-language version by a psychologist fluent in English. Then, an English philologist made the necessary linguistic corrections. The back translation was performed by five people, including three psychologists fluent in English and two philologists, ultimately obtaining a Polish version linguistically equivalent to the English one.

Next, a pilot study was conducted to ensure that native Polish speakers would be able to understand the content of the translated version accurately. The pilot study involved 24 university students. In the think-aloud session it was confirmed that the participants reported the intended meanings of the items. When analyzing the answers to the individual items, no floor or ceiling effects were observed. There was also no granularity of the response. The data from the pilot study were not included in the final analysis.

### Participants

The validation study (Study 1) was conducted online between January 17^th^ and March 16^th,^ 2020, with participants recruited via social media (Facebook, Instagram, Twitter). A guarantee of voluntary participation in the research was included in the invitation to the study, which was followed by informed consent. Only participants who read the invitation fully and gave consent were allowed to participate in the study. The protocol of the study was approved by the Scientific Research Ethics Committee at the University of Social Sciences and Humanities in Warsaw (application No. 50/2019/2), with a positive opinion (No. 2/2020) issued on January 14, 2020.

The final sample comprised 1651 participants aged 18–73 (80.62% females; M_age_ = 31.38, SD = 9.25). Among them, 1106 (67%) lived in a big city with more than 100,000 inhabitants, 727 (44.04%) had a master’s degree, 933 (56.51%) worked full-time, and 260 (15.75%) were still studying. A total of 1187 participants (71.9%) were in a stable relationship. Moreover, 755 (45.73%) had a good financial situation with a monthly net income from 4,500 PLN up to PLN 5,999 per capita in a household and 597 (36.16%) had an average financial situation with a monthly net income from 3,000 PLN up to PLN 4,499 per capita in a household. In total, 415 participants (25.14%) suffered from chronic disease.

The study to test stability over time (Study 2) was conducted online 10 months after the first measurement (from November 8^th^ 2020 to January 8^st^ 2021) among 344 participants (21% of the initial sample) aged 18–72 (66% females; M_age_ = 32.71; SD = 9.75) who had agreed in advance to be contacted for further measurements. Most of them, 202 (69.48%), lived in a big city with more than 100,000 inhabitants, and 152 (43.61%) had a master’s degree.

### Instrument of study

The SMM [4] consists of eight items in both versions (SMM-G, and SMM-S). The general version of the scale refers to stress in general, and the specific version is designed to measure mindset toward one specific source of stress. In the case of our study, a participant choose the most potent stress source in his/her current life. The participants were asked to express on a five-point Likert scale (5 = I definitely agree; 4 = I agree; 3 = I neither agree nor disagree; 2 = I disagree; 1 = I definitely disagree) the extent to which their experience was represented by each statement. Four items (1, 3, 5, 7) required reverse recording. For both the SSM-G and SMM-S, a higher summary score indicates a more enhancing stress mindset.

### Theoretical validity assessments

Other measures theoretically related to stress mindset were included to assess convergent and discriminant validity. These comprised measures of personality or personality-related traits, and perceived stress, as well as symptoms of depression and corresponded to the constructs used in previous validity research of the SMM.

#### Ten-Item Personality Inventory—TIPI (short version)

The TIPI [14; for the Polish adaptation, see 15] is the most popular, short method used to measure personality defined as the “Big Five” (neuroticism, extraversion, conscientiousness, openness to experience, and agreeableness) [16]. It consists of ten statements that begin with the words: *I see myself as a person*. Each subscale is represented by two items. The participants are asked to respond to each statement on a seven-point Likert scale (from 1 = strongly disagree, to 7 = strongly agree). An intensity of traits is described by the summed-up values for each subscale separately. Cronbach’s α for the tested samples was .70 in both studies.

#### Center for Epidemiologic Studies Depression Scale—CESD-R

The CESD-R [17; for the Polish adaptation, see 18] is a self-report scale consisting of 20 statements concerning symptoms of depression. The participants are asked to choose one of five possible answers regarding the frequency of the symptom. Responses are given on a scale from 0 = absent or less than one time per day; to 4 = almost daily for two weeks. A number of depressive symptoms is assessed on the basis on the summed results, with a higher value indicates higher symptomatology. Cronbach’s α for the tested samples was .94 in both studies.

#### Positive Orientation Scale—POS

The POS [19; for the Polish adaptation, see 20] measures the fundamental tendency to perceive and pay attention to positive aspects of life, one’s experience, and the self [21]. The positive orientation is an integrative higher-order latent construct indicated by three components: self-esteem, optimism, and life satisfaction [19, 20]. The POS contains eight statements to which the person responds on a five-point Likert scale (from 1 = I strongly disagree; to 5 = I strongly agree). The result is the sum of all the answers; the higher it is, the higher the level of positive orientation. Cronbach’s α for the tested samples was .89 (Study 1) and .88 (Study 2).

#### Ego Resiliency Scale—ERS

The ERS [22; for the Polish adaptation, see 23] measures ego-resiliency as a trait, defined as the ability to vary, in an adaptive manner, the degree to which one inhibits or expresses emotional impulses, depending on social demands. The ERS consists of fourteen items accompanied by a four-point Likert scale (from 1 = doesn’t relate at all; to 4 = strongly relates). The result is the sum of all the answers, except item number 10, which is not counted. The maximum score, 52 points, represents the highest level of resiliency. Cronbach’s α for the tested samples was .79 (Study 1) and .76 (Study 2).

#### Self-Control Scale—SCS

The SCS [24; for the Polish adaptation, see 25] measures self-control as a trait. This questionnaire consists of 36 items with response provided on a five-point Likert scale (from 1 = it doesn’t describe me at all; to 5 = it describes me very well). It includes five subscales: general self-discipline (11 items), prudent / non-impulse behavior (10 items), healthy habits (7 items), ethical behavior (5 items), and reliability (5 items). In the studies only the overall index was used. The general score is calculated by adding up the points and a higher values indicates a higher level of self-control. Cronbach’s α for the tested samples was .87 (Study 1) and .88 (Study 2).

#### Perceived Stress Scale (at Work)—PSS

The PSS (at Work) [26] is based on the PSS-10 [27]. The questionnaire is used to assess occupational stress, understood as assessing an employee’s adjustment to a work environment. It consists of 10 items expressed on a five-point Likert scale. The participants answer (from 1 = never; to 5 = always) how often they experience the states described in a particular item. Stress at work is calculated by summing up all the answers. Thus, a higher indicator describes a higher perceived stress. Cronbach’s α for the tested samples was .86 (Study 1) and .88 (Study 2).

#### WHOQL-BREF

The WHOQOL-BREF [28] is a shorter version of the WHOQOL-100. Both these tools were developed by the World Health Organization (WHO) and published in 1996. The WHOQOL-BREF measures quality of life, health and well-being. In our research, only one item of this scale was used (item number 2), containing a self-assessment question of one’s own health on a five-point Likert scale, from 1 = very dissatisfied; to 5 = very satisfied.

### Statistical analysis

The analysis consisted of four steps. First, to determine the amount and type of missing data; a missing values analysis (MVA) was performed using the IBM SPSS Statistics 27 statistical package and Version 4.0.5 of the R programming language [29]. The missing data were handled using the Bayesian imputation in IBM SPSS AMOS version 27.00 [30].

Next, exploratory (EFA), and confirmatory factor analysis (CFA) were performed to establish a factorial validity. In EFA, principal component analysis was used to extract factors, which were then orthogonally rotated with Varimax rotation. In CFA, the following five different models were tested: a single-factor model, a two-factor model and a four-factor model, a hierarchical model with four lower-order factors and one higher-order factor, and a bi-factor model with one general factor and correlated factors representing: 1 beliefs of stress consequences for health and vitality outcomes (Health and Vitality); 2 for performance and productivity outcomes (Productivity and Performance); and 3. for learning and growth outcomes (Learning and Growth). The following indices were used to evaluate a goodness of fit: TLI = Tucker-Lewis index (≥ .90), RMSEA = root mean square error of approximation (≤ .06), and CFI = comparative fit index (≥ .90) [31].

In the third step, reliability was evaluated in two approaches: as an internal reliability via Cronbach’s alpha and as over-time stability with a test-retest correlation coefficient. Finally, theoretical validity was assessed by examining a correlation pattern with other relevant constructs. To support a discriminant validity we expected that an absolute value of the correlations would be not higher than moderate (i.e., below |.60|). The analyses were performed using IBM SPSS Statistics 27 and IBM SPSS Amos, version 27.

## Results

### Missing data

Some missing data in Study 1 were noted for 349 participants, which accounts for 21.14% of the sample. The Little’s test result was statistically significant, χ2 (1.101) = 2,306.625; p < .001, thus missing data cannot be regarded as missing completely at random (MCAR). A series of independent samples *t*-tests and chi square test results turned out to be significant for several variables such as: POS t(77) = -8.06; p < .001; d = -1.35; TIPI agreeableness t(78) = 4.81; p < .001; d = .72; and TIPI emotional stability t(91) = 4.81; p < .001; d = -.85; as well as: place of living χ2 (3) = 130.21; p < .001; education χ2 (6) = 334.34; p < .001; professional status χ2 (6) = 346.78; p < .001; residence status χ2 (3) = 88; p < .001. Thus more missing data was observed for people living in small towns, without higher education, and unemployed.

Analogically, the missing data of Study 2 represented 15.4% of the database (51 participants) and did not follow the pattern of missing completely at random either. (Little’s test: χ2 (666) = 865.714; p < .001). The analysis showed significant differences for SMM-G, SMM-S and WHOQOL.

Thus, a Bayesian imputation was performed for handling missing data, separately for each study dataset. Further analyses were conducted on the data after this imputation.

### Descriptive statistics

The descriptive statistics for the two versions of SMM: SMM-G and SMM-S, as well as for other questionnaires used in the study are provided in Table 1 (Study 1 in the upper panel, Study 2 in the lower panel). As can be seen, the values in both studies are highly similar. The Kolmogorov-Smirnov test for all variables was significant. However, neither kurtosis nor skewness exceeded the conventional absolute value of 1.5 [32]. Additionally, the scores of all individual items of SMM-G and SMM-S (Table 2) had a normal distribution.

**Table 1:** Descriptive statistics of the variables with the Kolmogorov-Smirnov test.

Variable	*M*	*Mdn*	*SD*	*Skew*.	*Kurt*.	*Min*.	*Max*.
**Study 1**							
**SMM-G**	1.50	1.50	.72	.03	-.63	0	4
**SMM-S**	1.30	1.13	.77	.24	-.58	0	3.5
**POS**	3.52	3.63	.76	-.30	.52	1	6.13
**TIPIex**	4.88	5.00	1.51	-.54	-.46	1	7
**TIPIa**	5.28	5.50	1.25	-.73	.14	1	7
**TIPIc**	4.89	5.00	1.50	-.56	-.45	1	7
**TIPIes**	3.88	4.00	1.69	.04	-1.07	1	7
**TIPIoe**	4.77	5.00	1.26	-.32	-.22	.50	7
**CESD-R**	1.28	1.1	.88	.72	-.30	0	3.90
**PSS**	2.78	2.80	.74	.08	-.54	1	4.80
**ERS**	2.89	2.93	.48	-.29	-.17	1.29	4
**SCS**	3.14	3.17	18.30	-.08	-.15	1.64	4.67
**Study 2**							
**SMM-G**	1.41	1.38	.74	-.03	-.79	0	3.38
**SMM-S**	1.21	1.00	.81	.39	-.61	0	3.25
**POS**	3.45	3.50	.67	-.60	.22	1.38	4.88
**TIPIex**	4.86	5.00	1.49	-.49	-.53	1	7
**TIPIa**	5.35	5.50	1.21	-.53	-.03	1.5	7
**TIPIc**	4.81	5.00	1.50	-.41	-.87	1	7
**TIPIes**	3.79	3.05	1.67	-.02	-.99	1	7
**TIPIoe**	4.97	5.00	1.11	-.24	-.31	1.50	7
**CESD-R**	1.41	1.30	.91	.56	-.50	0	3.85
**PSS**	2.85	2.90	.79	-.07	-.42	1.86	4.00
**ERS**	2.95	2.93	.44	-.06	-.37	1.29	4
**SCS**	3.22	3.22	.51	-.21	-.04	1.56	4.27

Notes: *SD*, standard deviation; *M*, mean; *Mdn*, median; *Skew*., skewness; *Kurt*., kurtosis;, SMM-G, *the Stress Mindset Measure*-*General*; SMM-S, *the Stress Mindset Measure*-*Specific*; POS, *the Positivity Scale*; TIPI, short version of *the Ten-Item Personality Inventory*; TIPIex, TIPI extraversion subscale; TIPIa, TIPI agreeableness subscale; TIPIc, TIPI conscientiousness subscale; TIPIes, TIPI emotional stability subscale; TIPIoe, TIPI openness to experience, CESD-R, *the Center for Epidemiologic Studies Depression Scale—Revised*, PSS, *the Perceived Stress Scale at Work*; ERS, *the Ego Resiliency Scale*; SCS, *the Self-Control Scale*.

**Table 2:** Descriptive statistics for items of the *Stress Mindset Measure* (only Study 1): SMM-G and SMM-S.

Item no.	*M*	*Mdn*	*SD*	*Skewness*	*Kurtosis*
**SMM-G**					
1.r	1.50	1.00	1.00	.25	-.67
2.	1.72	2.00	1.09	.06	-1.13
3.r	1.00	1.00	.83	.89	.90
4.	1.82	2.00	1.09	.02	-1.12
5.r	1.77	2.00	1.08	.09	-1.03
6.	.85	1.00	.71	.83	1.59
7.r	1.69	1.00	1.08	.13	-1.06
8.	1.62	2.00	.87	-.03	-.42
**SMM-S**					
1.r	1.32	1.00	.96	.56	-.36
2.	1.40	1.00	1.05	.52	-.69
3.r	1.01	1.00	.88	.92	.67
4.	1.49	1.00	1.08	.41	-.86
5.r	1.53	1.00	1.09	.29	-.96
6.	.85	1.00	.72	.84	1.40
7.r	1.50	1.00	1.07	.38	-.86
8.	1.29	1.00	.95	.44	-.46

Notes: r, reversed item; *M*, mean; *Mdn*, median; *SD*, standard deviation; SMM-G, *the Stress Mindset Measure*-*General;* SMM-S, *the Stress Mindset Measure*-*Specific*.

### Exploratory factor analysis (EFA)

To test basic assumptions for EFA, the Kaiser-Meyer-Olkin (K-M-O) coefficient was calculated and Bartlett’s sphericity test was conducted. For the SMM-G the obtained values for Study 1 were KMO = .86 and Bartlett’s test χ2 (28) = 120519.12; p < .001; for Study 2 they were KMO = .85 and Bartlett’s test χ2 (28) = 23400.37; p < .001. The obtained values for the SMM-S for Study 1 were KMO = .90 and Bartlett’s test χ2 (28) = 144362.36; p < .001 and for Study 2 KMO = .86 and Bartlett’s test χ2 (28) = 30870.06; p < .001. Thus, the partial correlations between the items are high enough to indicate a significant overlap. Also, in each case the correlation matrix is not an identity matrix.

The EFA was then conducted for SMM-G and SMM-S for the data obtained in Study 1 and Study 2, separately. The sample from Study 1 (N = 1651) was additionally randomly divided into two subsamples, and the second one was used to verify the results obtained in first one.

The scree plot’s analysis suggested a two-factor distribution for the SMM-G and a single-factor distribution for the SMM-S for both subgroups. Moreover, eigenvalues higher than 1 supported the same number factors (Table 3). Thus, the following models were examined: for the SMM-G two-factor and for the SMM-S single-factor one.

**Table 3:** EFA results for the SMM-G and SMM-S: Initial Eigenvalues and total variance explained.

Variable	*F*	Subsample 1			Subsample 2		
		λ	*% of varian*.	*% cumul*.	λ	*% of varian*.	*% cumul*.
**SMM-G**	1	4.34	54.25	54.25	4.34	54.26	54.26
	2	1.05	13.12	67.37	1.03	12.93	67.18
**SMM-S**	1	4.93	61.60	61.60	4.95	61.85	61.85

Notes: *F*, factor; λ, eigenvalue; SMM-S, *the Stress Mindset Measure*-*Specific*; SMM-G, *the Stress Mindset Measure*-*General*; *varian*., explained variance; *cumulat*., explained cumulative variance.

For the SMM-G a two-factor solution was stablished. In both subsamples, the first subscale included items 2, 4, 5, 7, while the other one—items 1, 3, 6, with ambiguous assignment of item 8, which had loadings above a criterion value of .50 on both factors (see Table 4). For the SMM-S a single-factor structure was obtained, with loadings of all items above .65 and almost equal in both subsamples (Table 4).

**Table 4:** EFA results for the SMM-G and SMM-S: Factor loadings (Study 1).

Item no.	SMM-G				SMM-S	
	Subsample 1		Subsample 2		Subsample 1	Subsample 2
	1	2	1	2	1	1
1.	.36	**.66**	.37	**.64**	**.80**	**.80**
2.	**.75** ^a^	.32	**.76**	.31	**.84**	**.83**
3.	^b^	**.81**		**.82**	**.72**	**.73**
4.	**.84**		**.85**		**.78**	**.77**
5.	**.79**		**.78**		**.84**	**.84**
6.		**.82**		**.82**	**.66**	**.66**
7.	**.83**		**.82**		**.80**	**.80**
8.	**.53**	**.56**	**.52**	.**57**	**.83**	**.83**

Notes: ^a^Loadings above .50 are in bold.

^b^Loadings below .30 are not presented.

Analogical analyses were conducted for the data obtained in Study 2. The results revealed a two-factor structure for the SMM-G and a single-factor structure for the SMM-S. However, regarding the individual loadings of the SMM-G items, the results differed from those obtained in Study 1, leading to not replicated content of the subscales. The details for these analyses are provided in tables in the S1 File.

### Confirmatory factor analysis (CFA)

In CFA, the following models were tested: single-factor, two-factor, four-factor, and two more complex models: a bi-factor model with correlated subscale-specific factors and a hierarchical model with four lower order factor s and one higher order factor. The single-factor and two-factor models were analyzed to verify the results of EFA. The four-factor model consisted of factors grouping two items per each and describing a specific area of functioning. It was then transformed into a hierarchical model by adding one higher-order factor with all these lower factors loaded on it.

Table 5 summarizes the goodness of fit indices for all tested models. The best goodness of fit for the SMM-G was noted for the hierarchical four-factor model. The same model was confirmed for the SMM-S. In Study 2, the identical models were analyzed, and similar results were obtained. A minor difference was that in Study 2, the hierarchical four-factor model for the SMM-S had a slightly better fit than for the SMM-G, whereas the opposite was observed in Study 1.

**Table 5:** CFA fit indexes of the SMM factor structure models (Study 1 and Study 2).

	Model	^χ2^ *value*	*df*	RMSEA	LO90	HI90	CFI	TLI
**Study 1**								
**SMM-G**	Single-Factor	15457.2	14	.183	.180	.185	.872	.670
	Two-factor	9665.2	14	.144	.142	.078	.920	.794
	Four-factor	1002.3	9	.058	.055	.061	.991	.963
	Bi-Factor	910.3	6	.068	.064	.071	.992	.955
	Hierarchical	449.4	9	.038	.036	.042	.996	.985
**SMM-S**	Single-Factor	9986.9	14	.147	.144	.149	.937	.873
	Two-factor	7152.0	14	.124	.122	.127	.955	.909
	Four-factor	1487.9	9	.071	.068	.074	.991	.971
	Bi-Factor	1184.0	10	.077	.073	.081	.993	.965
	Hierarchical	576.6	10	.041	.039	.044	.996	.990
**Study 2**								
**SMM-G**	Single-Factor	1626.7	14	.135	.130	.141	.931	.862
	Two-factor	1338.1	14	.123	.117	.128	.943	.887
	Four-factor	1815.2	9	.179	.172	.186	.923	.760
	Bi-Factor	1534.6	6	.201	.193	.210	.935	.695
	Hierarchical	253.1	9	.066	.059	.073	.990	.968
**SMM-S**	Single-Factor	3958.7	14	.212	.206	.217	.879	.758
	Two-factor	2901.2	14	.181	.176	.187	.912	.823
	Four-factor	1308.3	9	.152	.145	.159	.960	.876
	Bi-Factor	1172.4	6	.176	.167	.184	.964	.833
	Hierarchical	210.3	10	.056	.050	.063	.994	.983

Notes: Extraction method–maximum likelihood, χ2, Chi-Square Statistic; *df*, Degrees of Freedom; RMSEA, root mean square error of approximation; CFI, comparative fit index; TLI, Tucker-Lewis index.

**Fig 1:**
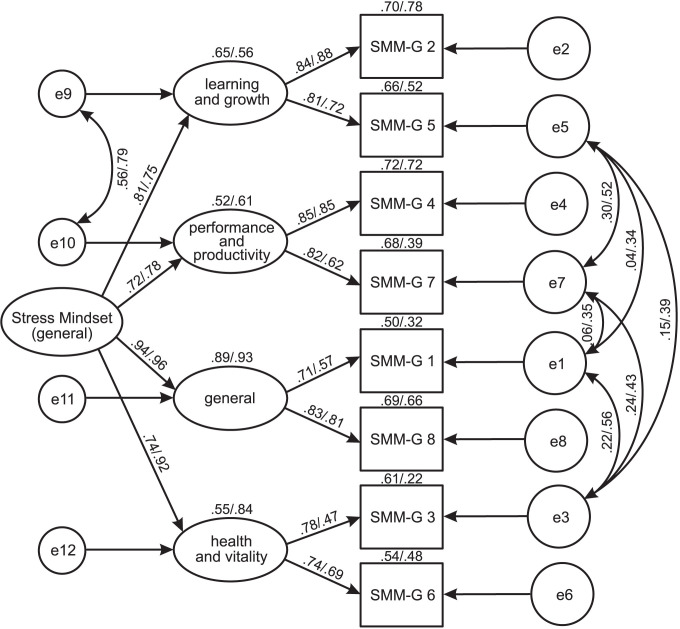
SMM-G: Results of CFA for a hierarchical four-factor model (Study 1 and Study 2, standardized solutions). SMM-G, *the Stress Mindset Measure-General*; SMM-G (1–8), items of a scale; Learning and Growth, subscale concerning beliefs of stress consequences for learning and growth; General, subscale concerning beliefs of stress consequences for overall life; Performance and Productivity, subscale concerning beliefs of stress consequences for performance and productivity; Health and Vitality, subscale concerning beliefs of stress consequences for health and vitality; e, residual.

**Fig 2:**
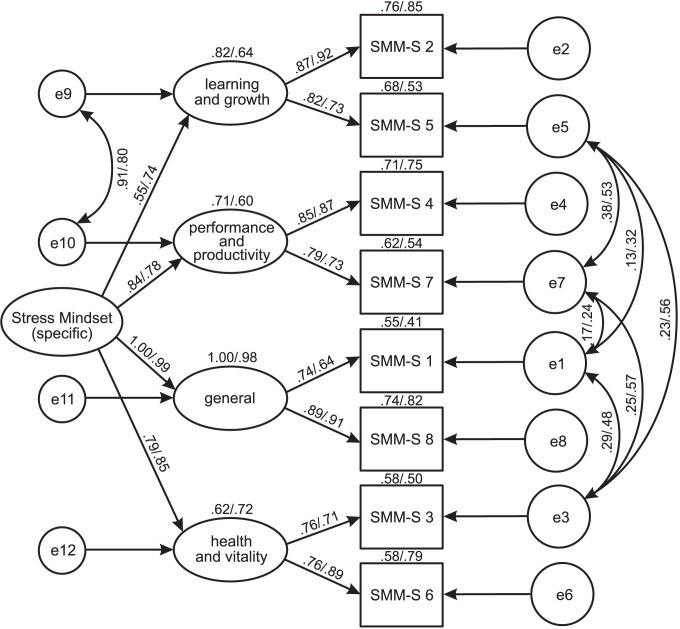
SMM-S: Results of CFA for a hierarchical four-factor model (Study 1 and Study 2, standardized solutions). SMM S, *the Stress Mindset Measure-Specific*; SMM-S (1–8), items of a scale; Learning and Growth, subscale concerning beliefs of stress consequences for learning and growth; General, subscale concerning beliefs of stress consequences for overall life; Performance and Productivity, subscale concerning beliefs of stress consequences for performance and productivity; Health and Vitality, subscale concerning beliefs of stress consequences for health and vitality; e, residual.

The resultants models are presented in Fig 1 (SMM-G in Study 1 and 2) and Fig 2 (SMM-S in Study 1 and 2). As can be seen, each lower-order factor covers two items containing beliefs of stress consequences for one functioning area, such as 1) Learning and Growth, 2) Performance and Productivity, 3) Health and Vitality, and 4) General. The item factor loadings for both versions of the SMM in Study 1 were higher than .70, and in Study 2—higher than .45, and half of them were above .90. In turn, the loadings of lower-order factors on the higher-order factor were within values from .72 to .96 in the SMM-G models (Studies 1 and 2) and within values from .55 to 1.00 in the SMM-S models.

The covariances between errors were placed only for items negatively worded, for which the raw scores were reversely coded [33]. The strength of these relations as well as their role in improving the goodness of fit of the models may point to the identification of an additional source of variance due to a specificity of a measurement method [34, 35].

In conclusion, the CFA provided empirical evidence of more complex than unidimensional structure of the SMM. Importantly, substantially the same hierarchical model was confirmed for both, general and specific version.

### Reliability

As presented in Table 6, the SMM-G had a good internal consistency for the overall index and subscales. However, these values varied across the subscales and across the samples. The lowest internal consistency was noted in Study 2 for Health and Vitality subscale of the SMM-G. For the SMM-S the analogical Cronbach’s alpha coefficients were higher. The correlation between two overall indices of the SMM-G was equal r = .62; *p* < .001, which can be regarded as a satisfactory stability taking into account the 10-month lag between the measurement points. Interestingly, this stability was only slightly lower for SMM-S, with a moderate correlation of r = .46, *p* < .001. Table 6 also shows the relevant correlations for each subscale.

**Table 6:** Reliability and stability over time (10 months) of the SMM.

Reliability Indices	Overall	Subscales			
		General	Health and Vitality	Performance and Productivity	Learning and Growth
**SMM-G**					
**Study 1: α**	.88	.73	.73	.82	.81
**Study 2: α**	.87	.76	.50	.70	.78
***r***	.62***^a^	.52***	.49***	.46***	.51***
**SMM-S**					
**Study 1: α**	.91	.79	.74	.80	.84
**Study 2: α**	.91	.74	.72	.77	.80
***r***	.46***	.41***	.39***	.35***	.41***

Notes: α, Cronbach’s alpha; *r*, Pearson correlation; Learning and Growth, subscale concerning beliefs of stress consequences for learning and growth; General, subscale concerning beliefs of stress consequences for overall life; Performance and Productivity, subscale concerning beliefs of stress consequences for performance and productivity; Health and Vitality, subscale concerning beliefs of stress consequences for health and vitality; SMM-G, *the Stress Mindset Measure*-*General*; SMM-S, *the Stress Mindset Measure*-*Specific*;

^a^*** *p* < .001.

### Theoretical validity

The theoretical validity analysis revealed that the SMM correlated in a predicted direction with measures of other constructs potentially important for cognitive, emotional and behavioral response to stressful situations. As Table 7 shows, correlations with emotional stability (TIPI), positive orientation (POS), ego-resiliency (ERS), and extraversion (TIPI) were weak and positive. Also, moderate, but negative correlations were noted with symptoms of depression (CESD-R) and perceived stress at work (PSS). The correlations of the SMM-G and SMM-S with openness to experiences (TIPI), self-control (SCS), and conscientiousness (TIPI) turned out to be positive and low. This indicates a lack of redundancy between the operationalizations of these constructs and stress mindset; thus, assessment of the stress mindset may indeed have an additional explanatory power in examining between-person differences in functioning under stress.

**Table 7:** Pearson correlation coefficients between SMM and other scales (Study 1 and Study 2).

Indicator	Study 1		Study 2	
	SMM-G	SMM-S	SMM-G	SMM-S
POS	.24*** ^a^	.23***	.25***	.33***
TIPIex	.20***	.16***	.25***	.22***
TIPIa	-.01	-.03***	-.08***	.01
TIPIc	.11***	.11***	.11***	.09***
TIPIes	.27***	.26***	.27***	.29***
TIPIoe	.19***	.12***	.19***	.23***
CESD-R	-.24***	-.31***	-.24***	-.36***
PSS	-.23***	-.28***	-.31***	-.29***
ERS	.25***	.17***	.30***	.28***
SCS	.15***	.18***	.19***	.15***
WHOQOLBREF	.18***	.24***	.13***	.23***

Notes: SMM-G, *the Stress Mindset Measure-General;* SMM-S, *the Stress Mindset Measure*-*Specific*; POS, *the Positivity Scale*; TIPI, short version of *the Ten-Item Personality Inventory*; TIPIex, TIPI extraversion subscale; TIPIa, TIPI agreeableness subscale; TIPIc, TIPI conscientiousness subscale; TIPIes, TIPI emotional stability subscale; TIPIoe, TIPI openness to experience; CESD-R, *the Center for Epidemiologic Studies Depression Scale—Revised*; PSS, *the Perceived Stress Scale at Work*; ERS, *the Ego Resiliency Scale*; SCS, *the Self-Control Scale;* WHOQOLBREF, short version of the WHOQOL-100;

^a^***, *p* < .001.

## Discussion

The current study aimed to establish psychometric properties of the *Stress Mindset Measure* in the Polish population. For factorial validity, the factor structure proposed for the original scale was not replicated. Instead, the hierarchical four-factor model turned out to be the best fitted to the data. Moreover, this structure was confirmed in the test-retest sample evaluated 10 months after the first measurement for both general and specific versions of the SMM. Thus, the following four lower-order factors were identified: 1) General (beliefs of stress consequences for overall life); 2) Health and Vitality (beliefs of stress consequences for health and vitality outcomes); 3) Productivity and Performance (beliefs of stress consequences for performance and productivity outcomes); 4) Learning and Growth (beliefs of stress consequences for learning and growth outcomes).

This factor structure gives rise to questioning the equivalence of the general lower-order subscale to the other three subscales. Firstly, it is an effect of highly unspecific meanings of items 1 and 8 that constitute this subscale (*The effects of stress are negative and should be avoided*, and *The effects of stress are positive and should be utilized*). The content of these items applies to stress in a very broad sense, in contrast to the other six items, which relate to the effects of stress in specific areas of an individual’s functioning (e.g., *Experiencing stress improves my health and vitality* or *Experiencing stress inhibits my learning and growth*). Secondly, in EFA, which was performed in two randomly selected numerically equal groups and in two studies, the loadings for item 8 behaved ambiguously, with changing factor affiliation depending on the data. This may indicate the superiority of items 1 and 8 over the others for measuring the general attitude toward stress, but this was not confirmed in the bi-factor model. Thus, it appears that with the present construction of the scale, the so-called general mindset is not an overriding factor but rather an unspecified-content factor.

The reliability of the obtained hierarchical four-factor solution was satisfactory in terms of both internal consistency (especially when taking into account the fact that there are only two items per subscale) and stability over time. However, it could be expected that for the SMM-G, values will be more coherent across subscales or perhaps with the highest values for the general “context-free” lower-order factor. The results also indicate that the SMM-G has good stability over time; but still, the common variance between repeated measurements in the same individuals is only about 41%. This suggests a spontaneous occurrence of within-person changes of stress mindset over a period of less than one year or imprecision of measurement. Therefore, it calls for further research.

The theoretical validity of the SMM was satisfactory, but mostly in terms of discriminant validity. This means that the SMM measures a construct that is only slightly similar to the measures of emotional stability, positivity, symptoms of depression self-assessment of health, perceived stress (at work) or ego- resiliency, but not the same. As such, this construct can therefore contribute to an explanation of incremental variance when analyzing inter-individual differences in stress response.

Interestingly, the hierarchical four-dimensional structure of the Polish validation of the SMM is consistent with the results of the psychometric analysis of the SCMM [12], where precisely the same lower-order factors were identified for the stress mindset. However, that result was obtained based on a set of more numerous and differently formulated items, which finally changed the operationalization of the stress mindset in comparison to that proposed by Crum and colleagues [4]. The major difference between the SMM and the SCMM lies in the wording of items. Namely, in the SMM, they described a *belief* towards stress, whereas in the SCMM the focus is on an individual’s ability to use the stress experience, so it is defined rather as a *skill* or *competence*. Thus, further research is required, especially as two somewhat different operationalizations of—declaratively—the same construct of stress mindset showed the same scale structure, which is, however, different from the original one obtained by the authors.

The current research is the first case of analysis of psychometric properties of the SMM in a sample of over 1500 participants, which is several times bigger than in the previous validations [4, 10, 11]. Thus, these results strongly add to the already existing findings [10, 11], not supporting a unidimensionality of the original English-language version. In this context, at least three non-excluding explanations are possible.

First, contextual factors may be more important for the stress mindset than previously assumed. It is essential to note that the original SMM’s validation study—as reported by its authors—consisted of participants who actively chose to be part of a stress management program, suggesting that they wanted to change their stress mindset. The well-known personality psychology question then arises about the relationship between the trait-like nature and the state-like nature of the stress mindset understood in terms of individual differences. Second, the operationalization itself may not be free from limitations, particularly as at the measurement level, it confuses specific stress mindsets with the more general or overall stress mindset as a meta-trait or meta-attitude. Another similar limitation is also visible in the unexpected method effect in CFA due to the negative wording of half of the items. Particularly, as discussed above, the SCMM shows that the final results may be very sensitive to the phrasing of items. Finally, the scale may be highly prone to culture differences thus its cross-culture construct validity calls for further research.

Additionally, our study is not free from limitations. It was conducted in a part of the digitally active population, mainly female, young, well-educated and from large cities. The self-reported prevalence of somatic diseases was also considerable. Furthermore, the missing data did not follow the pattern of missing completely at random. The results should therefore be treated with caution, especially regarding their generalizability.

## Conclusions

Based on the current study, it can be concluded that the Polish validation of the SMM in both the general and specific versions had good psychometric properties. However, it should be kept in mind that the obtained factor structure, although present in other validation studies, is substantially different from that obtained by the authors of the original. The extent and type of these discrepancies indicate a need to revise the proposed stress-mindset measure and contribute to questions about this construct’s theoretical nature.

## Supporting information

S1 File (DOCX)
