# Supplementary material for: Psychometric properties of the Stress Mindset Measure (SMM) in the Polish population
Source: PLoS One. 2022 Mar 22;17(3):e0264853. doi: 10.1371/journal.pone.0264853 (PMC8939810; doi:10.1371/journal.pone.0264853)
Supplement: S1 File — (DOCX) [file pone.0264853.s001.docx]

Supplementary Materials

**Psychometric properties of the *Stress Mindset Measure* (SMM)**

**in the Polish population**

Dorota Mierzejewska-Floreani^1*^, Mateusz Banaszkiewicz^1^, Ewa Gruszczyńska^1^

^1^SWPS University of Social Sciences and Humanities, Warsaw, Poland

**Table 1. EFA Results for the SMM-G and SMM-S: Factor Loadings (Study 2)**

| **Item no.** | **SMM-G** | | | | **SMM-S** | |
| --- | --- | --- | --- | --- | --- | --- |
|  | **Subsample 1** | | **Subsample 2** | | **Subsample 1** | **Subsample 2** |
|  | 1 | 2 | 1 | 2 | 1 | 1 |
| 1. | ^b^ | **.83** |  | .**82** | **.76** | **.77** |
| 2. | **.88**^a^ |  | **.87** |  | **.82** | **.83** |
| 3. |  | **.89** |  | .**90** | **.76** | **.74** |
| 4. | **.84** |  | **.82** |  | **.77** | **.76** |
| 5. | **.68** | .42 | **.61** | .52 | **.81** | **.81** |
| 6. | **.52** | .39 | .**54** | .32 | **.74** | **.71** |
| 7. | **.56** | .52 | .47 | **.65** | **.81** | **.82** |
| 8. | **.69** |  | .**72** |  | .**81** | .**81** |

Notes: ^a^Loadings above .50 are in bold. ^b^Loadings below .30 are not presented.
